# Performance Evaluation of Fixed Sample Entropy in Myographic Signals for Inspiratory Muscle Activity Estimation

**DOI:** 10.3390/e21020183

**Published:** 2019-02-15

**Authors:** Manuel Lozano-García, Luis Estrada, Raimon Jané

**Affiliations:** 1 Biomedical Signal Processing and Interpretation group, Institute for Bioengineering of Catalonia (IBEC), The Barcelona Institute of Science and Technology (BIST), UPC Campus Diagonal-Besòs, Av. d’Eduard Maristany 10–14, 08930 Barcelona, Spain; 2 Biomedical Research Networking Centre in Bioengineering, Biomaterials and Nanomedicine (CIBER-BBN), 08028 Barcelona, Spain; 3 Department of Automatic Control (ESAII), Universitat Politècnica de Catalunya (UPC)-Barcelona Tech, 08028 Barcelona, Spain

**Keywords:** electromyography, fixed sample entropy, mechanomyography, non-invasive physiological measurements, oesophageal electromyography, respiratory muscle

## Abstract

Fixed sample entropy (fSampEn) has been successfully applied to myographic signals for inspiratory muscle activity estimation, attenuating interference from cardiac activity. However, several values have been suggested for fSampEn parameters depending on the application, and there is no consensus standard for optimum values. This study aimed to perform a thorough evaluation of the performance of the most relevant fSampEn parameters in myographic respiratory signals, and to propose, for the first time, a set of optimal general fSampEn parameters for a proper estimation of inspiratory muscle activity. Different combinations of fSampEn parameters were used to calculate fSampEn in both non-invasive and the gold standard invasive myographic respiratory signals. All signals were recorded in a heterogeneous population of healthy subjects and chronic obstructive pulmonary disease patients during loaded breathing, thus allowing the performance of fSampEn to be evaluated for a variety of inspiratory muscle activation levels. The performance of fSampEn was assessed by means of the cross-covariance of fSampEn time-series and both mouth and transdiaphragmatic pressures generated by inspiratory muscles. A set of optimal general fSampEn parameters was proposed, allowing fSampEn of different subjects to be compared and contributing to improving the assessment of inspiratory muscle activity in health and disease.

## 1. Introduction

Measuring respiratory muscle function is a key step in the assessment of many respiratory diseases, such as chronic obstructive pulmonary disease (COPD) [1]. Respiratory muscle function is typically measured as pressure and lung volume changes [2]. Transdiaphragmatic pressure (P_di_) is the gold standard measure of the force exerted by the diaphragm, the main inspiratory muscle [3], and depends on the neural drive to the diaphragm and the level of diaphragm electrical activation. The electrical activity generated by the diaphragm can be accurately assessed by crural diaphragm electromyography (oesEMG_di_), using a multipair oesophageal electrode [4]. However, invasive measurement of P_di_ and oesEMG_di_ is technically complex and can be uncomfortable for study participants. Alternatively, inspiratory muscle force and activation can be measured by surface mechanomyography (MMG) [5,6] and electromyography (EMG) [6,7,8,9,10], respectively. The surface mechanomyogram is a non-invasive measure of muscle fibre vibration during muscle contraction and is considered to be the mechanical counterpart of motor unit electrical activity as measured by surface electromyography. Surface mechanomyogram and electromyogram of inspiratory muscles are typically measured using accelerometers and bipolar electrode pairs, respectively, positioned on the chest wall over the lower intercostal spaces (sMMG_lic_ and sEMG_lic_ respectively) [6] or the parasternal intercostal spaces (sMMG_para_ and sEMG_para_ respectively) [8].

Inspiratory muscle EMG and MMG signals are, however, contaminated by cardiac noise corresponding to the electrical (ECG) and mechanical (MCG) activity of the heart, respectively. Conventional approaches to analyse EMG and MMG signals, based on amplitude estimators, such as the average rectified value (ARV) or the root mean square (RMS) [11,12], are greatly influenced by cardiac activity. Therefore, an ARV- or RMS-based analysis of inspiratory muscle EMG and MMG signals implies prior rejection of signal segments that contain cardiac noise. This is a subjective and time-consuming task when performed manually. Furthermore, since the frequency content of cardiac noise overlaps greatly with that of inspiratory muscle EMG and MMG signals, it is difficult to reduce cardiac noise using conventional frequency filters. Although some automatic algorithms have been previously proposed to remove cardiac noise from EMG signals [13,14], these algorithms involve more complex filters or the recording of an extra ECG channel for QRS complexes detection.

As an alternative, our group has recently proposed fixed sample entropy (fSampEn) as a method to estimate respiratory muscle function from sEMG_lic_ [6,9] and sMMG_lic_ [6,15] signals, attenuating interference from cardiac activity. Based on sample entropy (SampEn) [16], fSampEn is a measure of regularity and complexity of time-series signals, so that more regular signals are less complex and lead to lower values of fSampEn. The following parameters must be fixed in fSampEn: the length of the time-series analysed, *N* (referred as window length), the length of the sequences to be compared, *m* (embedded dimension), and the tolerance for accepting that two sequences are similar, *r*. In SampEn, tolerance is set for each time-series as *r* times the standard deviation (SD) of the time-series analysed, so that SampEn is not influenced by differences in amplitude. However, fSampEn is intended to track both complexity and amplitude variations, and therefore it is determined by calculating SampEn using a fixed tolerance for different time-series. In this way, amplitude variations of a single time-series can be tracked by calculating fSampEn within a moving window and using a fixed tolerance of *r* times the SD of the whole time-series.

Therefore, fSampEn is not only sensitive to changes in signal complexity, but also to changes in signal amplitude [15]. In myographic respiratory signals, fSampEn has proven to be less sensitive in quantifying amplitude variations of more deterministic signal components, such as ECG and MCG, than in quantifying amplitude variations of more complex signal components, such as inspiratory muscle EMG and MMG [9,15]. Due to this advantageous property of fSampEn, this technique has been used in several applications related to respiratory muscles in healthy subjects, such as estimation of neural respiratory drive [9] and respiratory muscle activity [6,15,17] from inspiratory muscle EMG and MMG signals acquired during incremental loaded breathing, or estimation of neural inspiratory time onset and offset from inspiratory muscle EMG signals [18]. fSampEn has also been proposed to estimate inspiratory muscle mechanical activation efficiency from inspiratory muscle MMG signals acquired in COPD patients [19]. Furthermore, fSampEn has been used for the analysis of non-respiratory muscle activity [20,21,22,23,24].

Despite the potential for using fSampEn to analyse respiratory muscle EMG and MMG signals, there is no consensus standard for optimum fSampEn parameters, and several values have been suggested for window length, *m* and *r*. Previous studies [6,9,15,17,18,19,25] on respiratory muscles set *m* either at 1 or 2, *r* ranging from 0.1 to 0.5 and window length ranging from 0.25 to 1 s. A recent study evaluated the influence of window length, *m*, *r* and the sampling frequency on the estimation of respiratory activity from sEMG_lic_ signals using fSampEn [26], demonstrating that window length and *r* are the most critical parameters determining the shape and magnitude of fSampEn time-series. However, fSampEn time-series were calculated only for sEMG_lic_ signals recorded from one healthy subject and compared to non-invasive measurements of mouth pressure (P_mo_). Therefore, there is need for further research on the performance of fSampEn in different myographic respiratory signals related not only to the level of inspiratory muscle activation in healthy subjects, but also to that in patients with impaired respiratory mechanics.

The principal aim of the present study is, therefore, to provide an in-depth evaluation of the performance of fSampEn in myographic respiratory signals, which lead us to propose a set of optimal general fSampEn parameters for inspiratory muscle activity estimation. We analysed different combinations of window length and *r* parameters to ensure an optimal performance of fSampEn in oesEMG_di_, sEMG_lic_, sMMG_lic_, sEMG_para_ and sMMG_para_ signals recorded in healthy subjects and COPD patients.

## 2. Materials and Methods

### 2.1. Data Acquisition

Measurements of inspiratory muscle force and activation were obtained from twelve healthy subjects (six male, age 33 (30–39) years, body mass index 22.2 (20.6–24.2) kg/m^2^, forced expiratory volume in 1 second/forced vital capacity 81.9 (74.1–83.9)%), with no history of cardiorespiratory or neuromuscular disease, and from fourteen stable COPD patients (nine male, age 68 (65–72) years, body mass index 25.5 (19.4–28.0) kg/m^2^, forced expiratory volume in 1 second/forced vital capacity 38.2 (30.2–46.5)%). This study was approved by the NHS Health Research Authority (NRES Committee London–Dulwich 05/Q0703) and the experiments conformed to the standards of the Declaration of Helsinki. All subjects were fully informed of any risk associated with the study and provided their written consent before participation.

**Figure 1:**
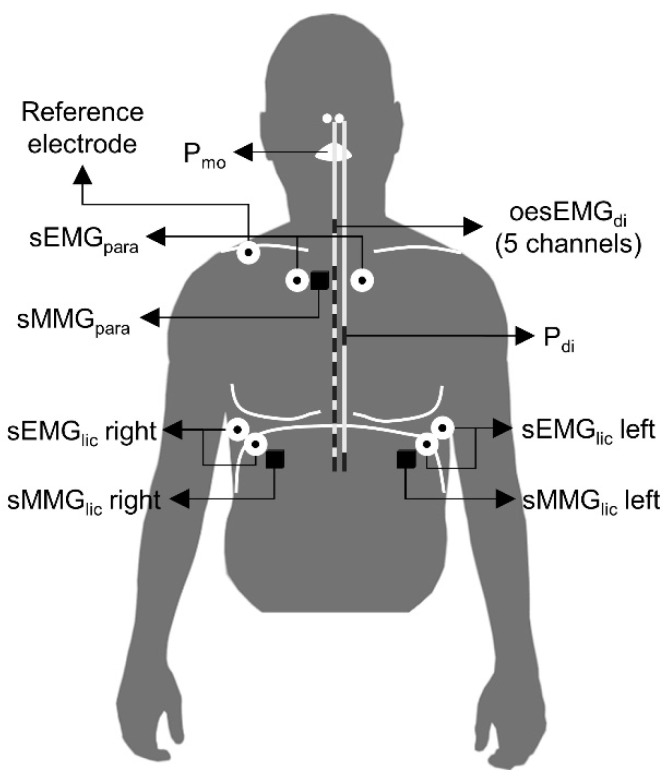
Sensors positioning for data acquisition. All signals were acquired in healthy subjects, but only P_mo_, sEMG_para_, sMMG_para_, sEMG_lic_ right and sMMG_lic_ right were recorded in COPD patients.

Non-invasive sEMG_lic_, sEMG_para_, sMMG_lic_, sMMG_para_, respiratory airflow and P_mo_ measurements were obtained from all participants (Figure 1). sEMG_lic_ was recorded bilaterally using two pairs of disposable surface Ag/AgCl electrodes (H124SG; Covidien Kendall) placed on the skin over the seventh or eighth intercostal spaces, between the mid-axillary and the anterior axillary lines [6,9,27]. sEMG_para_ was recorded using two surface electrodes positioned in the second intercostal space bilaterally [8,28]. A ground electrode was placed on the right clavicle. The skin was appropriately prepared prior to electrode application. sMMG_lic_ was recorded using two triaxial accelerometers (TSD109C2; BIOPAC Systems Inc, Goleta, CA, USA). The accelerometers were attached bilaterally to the skin with adhesive rings as close as possible to the sEMG_lic_ electrodes along the seventh or eighth intercostal space, over the anterior axillary line [6,19]. sMMG_para_ was recorded using another triaxial accelerometer placed on the right side in the second intercostal space, between the right sEMG_para_ electrode and the right border of the sternum. In COPD patients, sEMG_lic_ and sMMG_lic_ were recorded only on the right side for patients’ convenience. Respiratory airflow was measured using a pneumotachograph (4830; Hans Rudolph Inc, Shawnee, KS, USA) connected to a differential pressure transducer (DP45; Validyne Engineering, Northridge, CA, USA). P_mo_ was measured from a side port on the pneumotachograph using a second differential pressure transducer (MP45; Validyne Engineering).

Invasive P_di_ and oesEMG_di_ measurements were obtained from healthy subjects only (Figure 1), since these invasive tests can be uncomfortable for patients. P_di_ was measured as the difference between gastric and oesophageal pressures obtained using a dual-pressure transducer tipped catheter (CTO-2; Gaeltec Devices Ltd., Dunvegan, UK), as previously described [29,30]. Crural oesEMG_di_ was recorded using a multipair oesophageal electrode catheter (Yinghui Medical Equipment Technology Co. Ltd., Guangzhou, China), consisting of nine consecutive recording electrode coils, which formed five pairs of electrodes [1,31]. The pressure transducer and electrode catheters were inserted transnasally and once correctly positioned, taped to the nose to prevent movement during the study.

The EMG signals were amplified (gain 100), high-pass filtered at 10 Hz, and AC-coupled before acquisition (CED 1902; Cambridge Electronic Design Limited, Cambridge, UK). All signals were acquired using a 16-bit analogue-to-digital converter (PowerLab 16/35; ADInstruments Ltd., Oxford, UK) and displayed on a laptop computer running LabChart software (Version 7.2, ADInstruments Pty, Colorado Springs, CO, USA) with analogue to digital sampling at 100 Hz (airflow and pressures), 2000 Hz (MMG) and 4000 Hz (EMG).

### 2.2. Protocol

Maximal static inspiratory pressure (PImax) [2] was measured initially in all participants. This manoeuvre was repeated several times to ensure maximal volitional effort and each participant’s maximal PImax was used to determine the inspiratory threshold loads used in their individual incremental inspiratory threshold loading protocol.

All participants performed an inspiratory threshold loading protocol at five inspiratory threshold loads set at 12% (L1), 24% (L2), 36% (L3), 48% (L4) and 60% (L5) of the subject’s PImax. Inspiratory threshold loads were generated using an electronic inspiratory muscle trainer (POWERbreathe K5; POWERbreathe International Ltd., Southam, UK) attached to the distal end of the pneumotachograph. Subjects were seated and breathed through the pneumotachograph via a mouthpiece with a noseclip in place. Baseline measurements were recorded during a minimum of 2 minutes of quiet tidal breathing, following which the inspiratory muscle trainer was attached to the pneumotachograph and the series of threshold loads was imposed. Subjects were not provided with any specific instructions to adopt a certain duty cycle and were free to choose their own breathing frequency. Subjects were, however, informed that effort was needed to overcome the threshold loads, and they were therefore encouraged to focus on using their diaphragm, to perform quick deep inspirations and to ensure that expiration was complete before making their next inspiratory effort. Each load consisted of 30 breaths at most followed by a resting period to allow all respiratory measures to return to baseline.

### 2.3. Data Analysis

**Figure 2:**
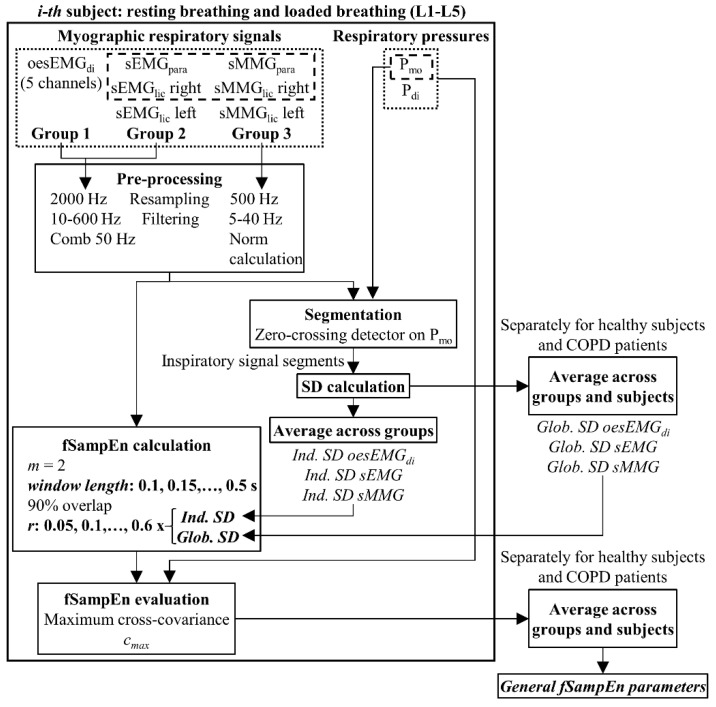
Data analysis block diagram. Dotted and dashed lines indicate signals/processes that were recorded/applied to healthy subjects and COPD patients, respectively. Ind.: individual; Glob.: global.

LabChart data were exported as MATLAB files, and analysed offline using our fSampEn algorithms developed in MATLAB (The MathWorks, Inc., vR2014a, Natick, MA, USA). Figure 2 shows a block diagram of the data analysis process described in the following sections.

#### 2.3.1. Pre-processing and Segmentation of Myographic Signals

oesEMG_di_, sEMG_lic_ and sEMG_para_ signals were resampled at 2000 Hz, and filtered with an 8th-order zero-phase Butterworth band-pass filter between 10 and 600 Hz and with a 2-Hz bandwidth notching comb filter to remove the power line interference at 50 Hz and all its harmonics up to 1000 Hz. Two 10th-order zero-phase notch filters were also applied to the EMG signals of COPD patients to remove additional interferences that appeared at 64 and 192.5 Hz. sMMG_lic_ and sMMG_para_ signals were resampled at 500 Hz and filtered with an 8th-order zero-phase Butterworth band-pass filter between 5 and 40 Hz. After filtering, the total acceleration measured by each accelerometer was arithmetically calculated as the norm of the vector formed by its three sMMG signals (sMMG_lic_ X, sMMG_lic_ Y and sMMG_lic_ Z for sMMG_lic_, and sMMG_para_ X, sMMG_para_ Y and sMMG_para_ Z for sMMG_para_).

All signals were segmented into inspiratory and expiratory signal segments by means of a zero-crossing detector on the P_mo_ signal. After segmentation, all cycles were visually inspected and those either containing artefacts within the EMG and MMG signals or having an unusual pressure pattern were rejected.

#### 2.3.2. Individual and Global SD Calculation

In order to track amplitude changes evoked by inspiratory muscle EMG and MMG activity during the inspiratory threshold loading protocol, fSampEn requires a single tolerance value to be fixed for each subject and group of myographic respiratory signals. Based on signal nature, the following three groups of signals were defined for each subject: one containing the five oesEMG_di_ signals (only for healthy subjects), another containing sEMG_para_ and right and left sEMG_lic_ signals (only right for COPD patients), and one more containing |sMMG_para_| and right and left |sMMG_lic_| signals (only right for COPD patients).

Since tolerance is usually set as *r* times the SD of the signal analysed, a unique individual SD was calculated for each subject and group of signals. Firstly, SD of all inspiratory signal segments during resting breathing and threshold loading was calculated for each signal. Then, SD values of the five oesEMG_di_ signals of each healthy subject were averaged to obtain a unique individual SD oesEMG_di_. In the same way, SD values of sEMG_para_ and right and left sEMG_lic_ signals (only right for COPD patients) of each subject were averaged to obtain a unique individual SD sEMG. Finally, SD values of |sMMG_para_| and right and left |sMMG_lic_| signals (only right for COPD patients) of each subject were averaged to obtain a unique individual SD |sMMG|. In this way, fSampEn time-series of a group of signals were calculated using the same tolerance value for all signals and all moving windows throughout resting breathing and the inspiratory threshold loading protocol.

It seems clear that, for a given subject and group of signals, a unique individual SD is required to make fSampEn time-series of resting breathing and threshold loading comparable. Moreover, a unique global SD is required in order to compare fSampEn time-series of different subjects. The question is, however, whether a global SD represents well the variation of a given group of signals in all subjects. To study the effect of using a global SD on fSampEn time-series, a unique global SD was calculated for each group of signals and separately for healthy subjects and COPD patients, as the mean of individual SDs.

#### 2.3.3. fSampEn Time-Series Calculation and Evaluation

For each subject, fSampEn time-series of all myographic respiratory signals (five oesEMG_di_, three sEMG_lic_ and three sMMG_lic_ in healthy subjects, and two sEMG_lic_ and two sMMG_lic_ in COPD patients) acquired during resting breathing and the inspiratory threshold loading protocol were calculated using *m* equal to 2, window length ranging from 0.1 to 0.5 s in increments of 0.05 s and tolerance set as *r* times SD, with *r* ranging from 0.05 to 0.6 in increments of 0.05 and SD equal to individual SD and global SD. A 90% overlap between adjacent windows was used. As a result, a total of 14,256 and 5184 fSampEn time-series were calculated for each healthy subject and each COPD patient, respectively.

fSampEn time-series were evaluated based on their similarity with P_mo_ and P_di_ in healthy subjects and with P_mo_ in COPD patients. Since fSampEn time-series and pressure signals have non-zero mean, similarity was calculated as the maximum cross-covariance, *c_max_*, of fSampEn time-series and pressure signals.

## 3. Results

### 3.1. Individual and Global SDs

**Figure 3:**
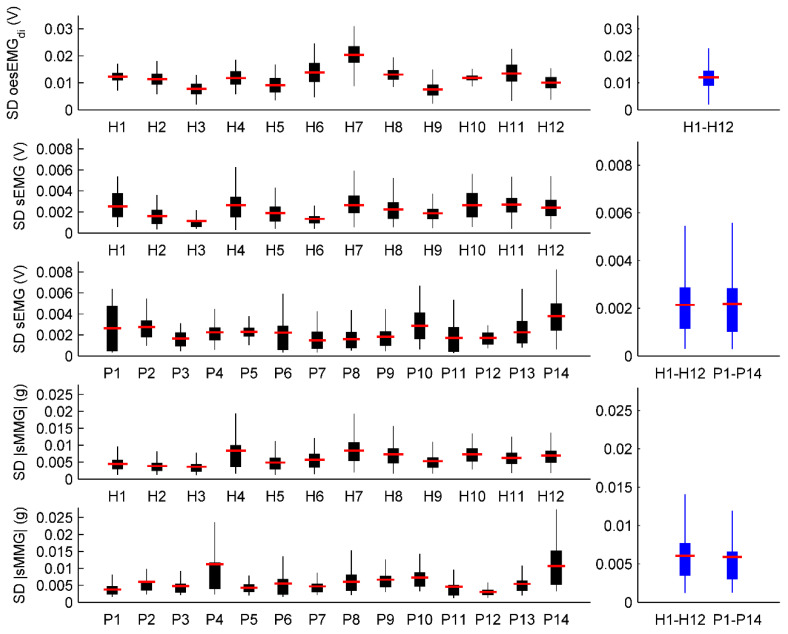
Boxplot distributions of individual SDs (H for healthy subjects and P for patients) are shown using black boxes. Red lines represent unique individual SDs (mean value). Boxplot distributions of global SDs are shown using blue boxes. Red lines represent unique global SDs (mean value).

Individual and global SDs are shown in Figure 3, for both healthy subjects and COPD patients, as well as for the three groups of signals (oesEMG_di_, sEMG and |sMMG|).

Interestingly, despite the intra- and inter-subject variability of individual SDs (black boxes), global SDs (blue boxes) of healthy subjects were very similar to those of COPD patients, for both sEMG (0.0021 V and 0.0022 V, respectively) and |sMMG| (0.0060 g and 0.0059 g, respectively) signals. A global SD oesEMG_di_ was calculated only for healthy subjects (0.0121 V). The effect of using a global instead of individual SDs is analysed in the next two sections, separately for healthy subjects and COPD patients.

### 3.2. Performance of fSampEn in Healthy Subjects

**Figure 4:**
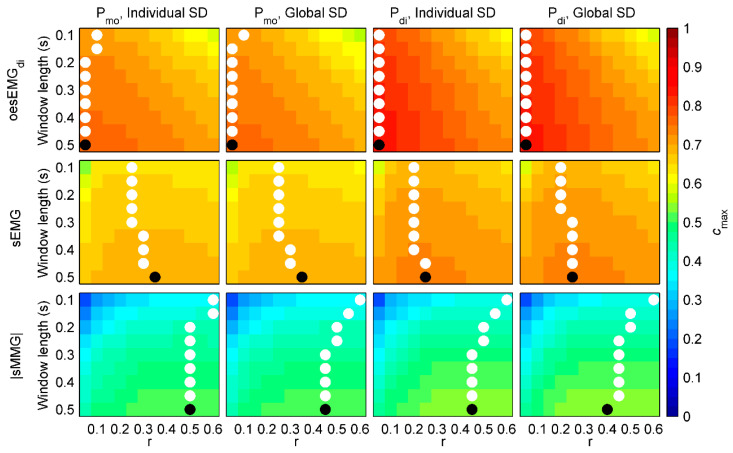
Similarity (*c_max_*) between fSampEn time-series of inspiratory muscle myographic signals (oesEMG_di_, sEMG and |sMMG|) and pressure signals (P_mo_ and P_di_) in healthy subjects. For each comparison, different values for window length (from 0.1 to 0.5 s), *r* (from 0.05 to 0.6) and SD (individual or global) were tested. White dots indicate the location of the highest *c_max_* of each row. Black dots indicate the location of the highest *c_max_* of the whole matrix.

Figure 4 shows *c_max_* values for healthy subjects. First, mean *c_max_* of each group of signals during resting breathing and threshold loading was calculated for each subject and combination of fSampEn parameters (window length, *r* and SD). Then, mean *c_max_* values of all healthy subjects were averaged.

Very similar *c_max_* values were obtained for oesEMG_di_, sEMG and |sMMG| using individual and global SDs, suggesting that global SDs could reasonably be used in order to make fSampEn time-series of different subjects comparable.

Using global SDs, the highest *c_max_* values in all cases were obtained for a global window length of 0.5 s. However, optimal global *r* varied among groups of signals: 0.05 for oesEMG_di_, 0.35 and 0.25 for sEMG, and 0.45 and 0.4 for |sMMG|.

The effect of using global instead of individual fSampEn parameters was measured for each healthy subject as the absolute difference, Δ*c_max_*, between the *c_max_* value obtained using global parameters and that obtained using individual parameters (Table 1, Table 2 and Table 3). Δ*c_max_* values were expressed as percentages of the *c_max_* value obtained using individual parameters.

Median Δ*c_max_* were below 3% for all groups of signals, indicating that the performance of fSampEn in healthy subjects is not affected using global instead of individual fSampEn parameters.

### 3.3. Performance of fSampEn in COPD Patients

**Figure 5:**
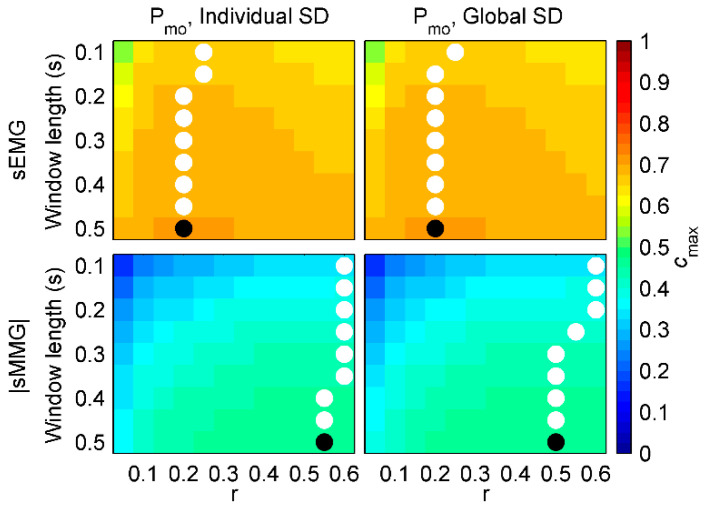
Similarity (*c_max_*) between fSampEn time-series of inspiratory muscle myographic signals (sEMG and |sMMG|) and pressure signals (P_mo_) in COPD patients. For each comparison, different values for window length (from 0.1 to 0.5 s), *r* (from 0.05 to 0.6) and SD (individual or global) were tested. White dots indicate the location of the highest *c_max_* of each row. Black dots indicate the location of the highest *c_max_* of the whole matrix.

Figure 5 shows *c_max_* values for COPD patients. First, mean *c_max_* of each group of signals during resting breathing and threshold loading was calculated for each patient and combination of fSampEn parameters (window length, *r* and SD). Then, mean *c_max_* values of all COPD patients were averaged.

As in healthy subjects, very similar *c_max_* values were obtained for sEMG and |sMMG| using individual and global SDs in COPD patients. Using global SDs, the highest *c_max_* values in all cases were obtained, again, for a global window length of 0.5 s. Regarding optimal global *r*, it varied from 0.2 for sEMG to 0.55 and 0.5 for |sMMG|.

The effect of using global instead of individual fSampEn parameters was measured for each COPD patient as the difference between the *c_max_* value obtained using global parameters and that obtained using individual parameters (Table 4).

In COPD patients, median Δ*c_max_* were below 1% for both sEMG and |sMMG| groups of signals, indicating that the performance of fSampEn in these patients is not affected using global instead of individual fSampEn parameters.

### 3.4. General fSampEn Parameters

In light of the very similar global SDs in healthy subjects and COPD patients (Figure 3), and the robustness of fSampEn to the use of global instead of individual fSampEn parameters, the following general fSampEn parameters are proposed to be used in healthy subjects as well as in COPD patients: 0.5 s moving window with 90% overlap, *m* equal to 2 and tolerance equal to 0.05 × 0.0121 (oesEMG_di_), 0.3 × 0.0022 (sEMG) and 0.5 × 0.0060 (|sMMG|).

**Figure 6:**
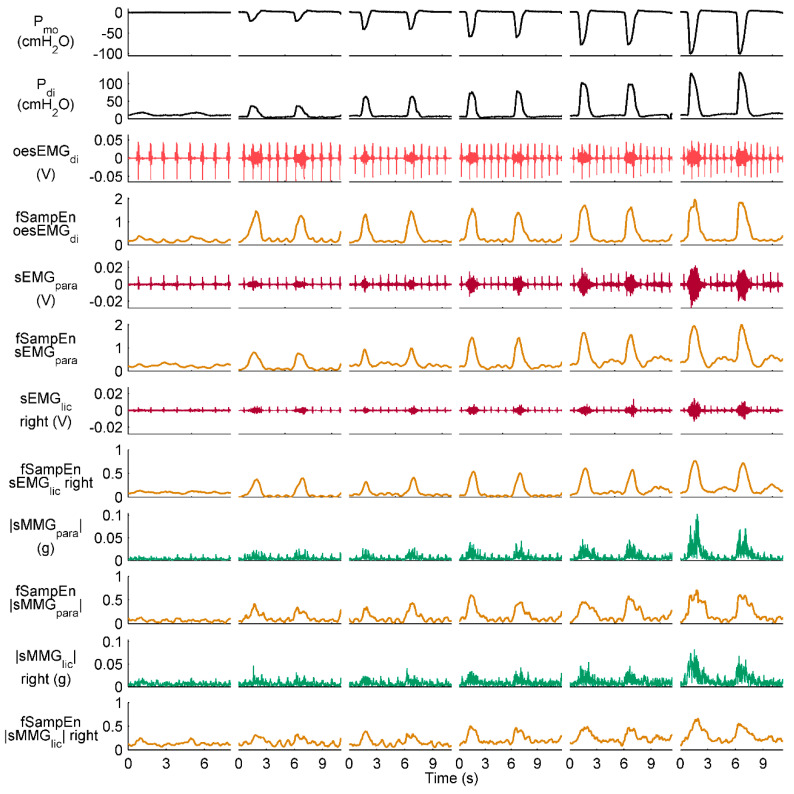
Measurements recorded during the inspiratory threshold loading protocol in a healthy subject. Two respiratory cycles are shown for quiet breathing and threshold loading. The oesEMG_di_ signal corresponds to the electrode pair 1. fSampEn time-series of the oesEMG_di_, sEMG and |sMMG| signals were calculated using the general fSampEn parameters proposed in this section.

**Figure 7:**
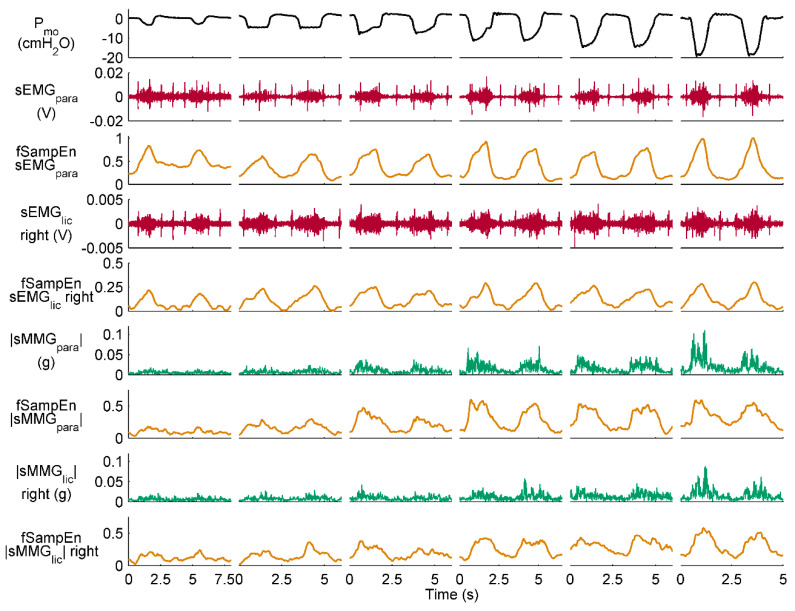
Measurements recorded during the inspiratory threshold loading protocol in a COPD patient. Two respiratory cycles are shown for quiet breathing and threshold loading. fSampEn time-series of the sEMG and |sMMG| signals were calculated using the general fSampEn parameters proposed in this section.

Figure 6 and Figure 7 show representative recordings from a healthy subject and a COPD patient, respectively, during resting breathing and the inspiratory threshold loading protocol. fSampEn time-series of these representative subjects’ myographic signals were calculated using the proposed general fSampEn parameters.

As observed in Figure 6 and Figure 7, fSampEn performed very well in all myographic signals, tracking amplitude changes evoked by inspiratory muscle EMG and MMG activity during the inspiratory threshold loading protocol as well as being robust to cardiac noise. 

## 4. Discussion and Conclusions

In this study, we conducted an evaluation of the performance of fSampEn for inspiratory muscle EMG and MMG analysis, which led us to propose, for the first time, a set of optimal general fSampEn parameters adapted to each type of myographic signal.

Evaluating the amplitude of myographic signals recorded from different respiratory muscles using conventional amplitude estimators is difficult due to the cardiac muscle interference. In order to reduce crosstalk from the heart in myographic respiratory signals, the use of fSampEn was adopted in our previous studies [6,9,15,17,19]. This technique is based on SampEn, which has previously contributed to improving the understanding of the underlying mechanisms of physiological processes in a wide number of clinical applications [16,32,33,34].

Depending on the application, fSampEn requires an adjustment of its input parameters *m*, *r* and window length. However, even for the same application, such as respiratory muscle activity estimation, several values have been suggested for fSampEn parameters. A previous study by our group demonstrated that *r* and window length are the most critical parameters, influencing the magnitude and shape of fSampEn time-series [26]. It was reported that a window length of 1 s and either *m* equal to 1 and *r* ranging from 0.1 to 0.64 or *m* equal to 2 and *r* ranging from 0.13 to 0.45 could be suitable values for respiratory muscle activity estimation using fSampEn in sEMG_lic_ signals. However, some limitations were acknowledged in this previous study. Firstly, only sEMG_lic_ signals were analysed for a healthy subject. However, other myographic signals, such as sMMG_lic_ or oesEMG_di_, could require different fSampEn parameters. Moreover, impaired respiratory mechanics (e.g., in COPD patients) may affect the amplitude and complexity of myographic respiratory signals [1,5,35], and therefore fSampEn parameters different to those used in healthy subjects may be required. Furthermore, the performance of different combinations of fSampEn parameters was evaluated by means of Pearson’s correlation coefficient between fSampEn time-series and P_mo_, which was used as an approximation of the overall mechanical output of the inspiratory muscles during breathing. However, P_mo_ was conditioned by the threshold loads imposed by the inspiratory muscle trainer, which was attached to the distal end of the pneumotachograph in series with a mouthpiece.

In this current study, we have thoroughly investigated the performance of fSampEn, for the first time, in different types of myographic respiratory signals, including oesEMG_di_, sEMG_lic_, sEMG_para_, sMMG_lic_ and sMMG_para_. Moreover, the participants in the present study cohort were twelve healthy subjects with an age range from 21 to 44 years and fourteen COPD patients with an age range from 53 to 80 years. This heterogeneous population covers a wide age range and both normal and impaired respiratory mechanics. Furthermore, the inspiratory threshold loading protocol provided the potential to acquire inspiratory muscle EMG and MMG signals under a wide range of loads on the inspiratory muscles, allowing the performance of fSampEn to be evaluated for a variety of inspiratory muscle activation levels. The performance of fSampEn was assessed by means of the cross-covariance of fSampEn time-series and both non-invasive measurements of P_mo_ and the gold standard invasive measurements of P_di_. While P_mo_ assesses global inspiratory muscle strength, P_di_ is more specific for the diaphragm.

The kernel of the fSampEn algorithm is the search for similar sequences within a signal, so that more similar sequences indicate lower complexity and yield lower fSampEn values. Two sequences are similar if the maximum difference of their corresponding values is less than or equal to the tolerance parameter, which is usually set as *r* times the SD of the signal analysed. When calculated within a moving window using a fixed tolerance for all windows, fSampEn can quantify amplitude variations on a signal. In this study, for a given subject and type of myographic respiratory signal, choosing a unique individual tolerance, and therefore a unique individual SD, allows fSampEn to track amplitude variations during the inspiratory threshold loading protocol and optimises the performance of fSampEn, mainly in terms of cardiac noise attenuation, for that subject. On the other hand, a unique global SD is required in order to compare fSampEn time-series of different subjects. Due to differences in myographic respiratory signal amplitude among subjects, using a global SD, however, may not be optimal in all subjects and may compromise the performance of fSampEn in some subjects.

In the present study we compared, for the first time, the performance of fSampEn in three groups of myographic respiratory signals (oesEMG_di_, sEMG and |sMMG|) using different *r* values (0.05–0.6) and both individual and global SDs. Intra- and inter-subject variability of the SD of myographic respiratory signals was firstly analysed (see Figure 3). Intra-subject variability was due to different levels of inspiratory muscle activation during the inspiratory threshold loading protocol. Inter-subject variability, however, was related to different patterns of inspiratory muscle activation during the inspiratory threshold loading protocol, since subjects were not provided with any specific instructions to adopt a certain breathing pattern. Moreover, inspiratory muscle EMG and MMG signals were not normalised, and therefore myographic signal amplitudes varied among subjects. Despite the inter-subject variability of SDs, individual and global SDs performed similarly in both healthy subjects and COPD patients. Moreover, global SDs of healthy subjects were very similar to those of COPD patients, for both sEMG and |sMMG| signals.

In light of these results, we have proposed general tolerance (*r* × SD) values of 0.05 × 0.0121 (oesEMG_di_), 0.3 × 0.0022 (sEMG) and 0.5 × 0.0060 (|sMMG|) to be used in healthy subjects as well as in COPD patients. The use of these general SDs allows fSampEn time-series to be optimally calculated and compared in different subjects.

Different combinations of window lengths between 0.1 s and 0.5 s were tested, and a window length of 0.5 s maximised the performance of fSampEn in all study participants and myographic respiratory signals. In general, the longer the window, the smoother the fSampEn time-series [26]. Long windows are therefore desirable to minimise the influence of cardiac noise or short-time variations of myographic respiratory signals, e.g., in neural respiratory drive estimation [9] or in inspiratory muscle activity estimation from the area under the curve of inspiratory EMG and MMG signals [6]. Conversely, short windows are required to evaluate short-time variations of myographic respiratory signals, e.g., in onset estimation of the neural inspiratory time [18].

In this study, fSampEn time-series were compared with pressure signals, which are low-frequency signals and vary slowly over time, and therefore longer windows performed better than shorter windows. On the other hand, a lower threshold of 0.5 s was applied to respiratory phase durations during segmentation of myographic signals. Therefore, in order to focus the analysis of myographic respiratory signals on inspiratory activity, window lengths longer than 0.5 s were not tested. Yentes et al. proposed a minimum window length of 200 samples for the correct performance of fSampEn [36]. The window length value of 0.5 s proposed in this study meets this criterion, even for |sMMG| signals (500 Hz x 0.5 s = 250 samples).

The *m* parameter is typically set at 1 or 2 [16,37]. In recent studies by our group, no relevant differences were found between *m* = 1 and *m* = 2 for fSampEn calculation in sEMG_lic_ signals [26], and *m* = 2 has been properly used for the analysis of different myographic respiratory signals [6]. Accordingly, *m* = 2 has also been used in the present study for the analysis of myographic respiratory signals.

Sampling frequency is another parameter to be taken into account in fSampEn calculation. It has been reported that higher sampling frequencies yield lower fSampEn values [26]. However, the effect of sampling frequency in fSampEn time-series can be omitted if sampling frequency is correctly chosen based on the effective bandwidth of each type of myographic respiratory signal, as in the present study.

In summary, we performed a thorough evaluation of the most relevant parameters of fSampEn, that are *r* and window length, for a proper estimation of inspiratory muscle activity from EMG and MMG signals. The evaluation of fSampEn performed in a heterogeneous study population, including healthy subjects and COPD patients within a wide age range, allowed us to propose general values for fSampEn parameters that permit optimal calculation and comparison of fSampEn time-series of different subjects, thus contributing to improving the assessment of inspiratory muscle activity in health and disease.

## Figures and Tables

**Table 1:** Difference between individual and global fSampEn parameters in the cross-covariance of oesEMG_di_ fSampEn time-series and pressure signals of healthy subjects.

Subject	oesEMG_di_ vs. P_mo_					oesEMG_di_ vs. P_di_				
	Ind. SD		Glob. SD		Δ*c_max_* (%)	Ind. SD		Glob. SD		Δ*c_max_* (%)
	Ind. *r*	*c_max_*	Glob. *r*	*c_max_*		Ind. *r*	*c_max_*	Glob. *r*	*c_max_*	
H1	0.20	0.767	0.05	0.758	1.15	0.05	0.869	0.05	0.870	0.06
H2	0.05	0.690	0.05	0.691	0.22	0.05	0.750	0.05	0.751	0.08
H3	0.10	0.776	0.05	0.775	0.09	0.05	0.782	0.05	0.779	0.32
H4	0.20	0.751	0.05	0.742	1.18	0.10	0.879	0.05	0.871	0.84
H5	0.40	0.723	0.05	0.691	4.53	0.05	0.883	0.05	0.883	0.05
H6	0.10	0.823	0.05	0.818	0.60	0.05	0.784	0.05	0.784	0.03
H7	0.25	0.743	0.05	0.725	2.36	0.05	0.941	0.05	0.942	0.06
H8	0.05	0.814	0.05	0.814	0.05	0.05	0.907	0.05	0.909	0.18
H9	0.05	0.799	0.05	0.798	0.10	0.05	0.848	0.05	0.845	0.43
H10	0.15	0.822	0.05	0.811	1.27	0.05	0.860	0.05	0.860	0.00
H11	0.30	0.726	0.05	0.716	1.32	0.05	0.868	0.05	0.869	0.10
H12	0.05	0.774	0.05	0.772	0.28	0.05	0.728	0.05	0.725	0.42
Median (IQR)	0.13 (0.05–0.21)	0.770 (0.739–0.803)	0.05 (0.05–0.05)	0.765 (0.723–0.801)	0.88 (0.19–1.28)	0.05 (0.05–0.05)	0.864 (0.784–0.880)	0.05 (0.05–0.05)	0.865 (0.783–0.874)	0.09 (0.06–0.35)

Ind.: Individual; Glob.: Global.

**Table 2:** Difference between individual and global fSampEn parameters in the cross-covariance of sEMG fSampEn time-series and pressure signals of healthy subjects.

Subject	sEMG vs. P_mo_					sEMG vs. P_di_				
	Ind. SD		Glob. SD		Δ*c_max_* (%)	Ind. SD		Glob. SD		Δ*c_max_* (%)
	Ind. *r*	*c_max_*	Glob. *r*	*c_max_*		Ind. *r*	*c_max_*	Glob. *r*	*c_max_*	
H1	0.60	0.765	0.35	0.747	2.32	0.40	0.850	0.25	0.829	2.54
H2	0.60	0.575	0.35	0.572	0.47	0.60	0.639	0.25	0.619	3.18
H3	0.60	0.663	0.35	0.664	0.12	0.60	0.658	0.25	0.653	0.80
H4	0.30	0.666	0.35	0.666	0.07	0.30	0.647	0.25	0.643	0.72
H5	0.60	0.707	0.35	0.703	0.57	0.35	0.801	0.25	0.800	0.04
H6	0.35	0.736	0.35	0.732	0.63	0.30	0.697	0.25	0.696	0.18
H7	0.50	0.714	0.35	0.706	1.12	0.20	0.842	0.25	0.842	0.00
H8	0.30	0.822	0.35	0.822	0.01	0.30	0.864	0.25	0.862	0.18
H9	0.10	0.709	0.35	0.689	2.69	0.15	0.720	0.25	0.718	0.35
H10	0.20	0.779	0.35	0.779	0.10	0.10	0.840	0.25	0.834	0.65
H11	0.45	0.585	0.35	0.568	2.87	0.25	0.701	0.25	0.699	0.27
H12	0.10	0.680	0.35	0.606	11.01	0.10	0.643	0.25	0.604	5.97
Median (IQR)	0.40 (0.28–0.60)	0.708 (0.665–0.744)	0.35 (0.35–0.35)	0.696 (0.649–0.736)	0.60 (0.11–2.41)	0.30 (0.19–0.36)	0.711 (0.655–0.840)	0.25 (0.25–0.25)	0.708 (0.650–0.830)	0.50 (0.18–1.23)

Ind.: Individual; Glob.: Global.

**Table 3:** Difference between individual and global fSampEn parameters in the cross-covariance of |sMMG| fSampEn time-series and pressure signals of healthy subjects.

Subject	|sMMG| vs. P_mo_					|sMMG| vs. P_di_				
	Ind. SD		Glob. SD		Δ*c_max_* (%)	Ind. SD		Glob. SD		Δ*c_max_* (%)
	Ind. *r*	*c_max_*	Glob. *r*	*c_max_*		Ind. *r*	*c_max_*	Glob. *r*	*c_max_*	
H1	0.55	0.642	0.45	0.641	0.10	0.50	0.649	0.40	0.648	0.13
H2	0.50	0.388	0.45	0.385	0.62	0.50	0.431	0.40	0.427	0.91
H3	0.50	0.443	0.45	0.439	0.94	0.60	0.431	0.40	0.432	0.21
H4	0.55	0.394	0.45	0.392	0.52	0.25	0.471	0.40	0.469	0.51
H5	0.55	0.529	0.45	0.529	0.02	0.50	0.562	0.40	0.562	0.03
H6	0.40	0.532	0.45	0.530	0.20	0.50	0.480	0.40	0.479	0.16
H7	0.15	0.530	0.45	0.524	1.29	0.20	0.653	0.40	0.650	0.33
H8	0.60	0.666	0.45	0.652	2.02	0.60	0.717	0.40	0.700	2.39
H9	0.45	0.470	0.45	0.469	0.24	0.50	0.478	0.40	0.478	0.06
H10	0.40	0.654	0.45	0.654	0.01	0.35	0.690	0.40	0.690	0.05
H11	0.55	0.536	0.45	0.532	0.62	0.50	0.642	0.40	0.639	0.47
H12	0.50	0.471	0.45	0.468	0.62	0.10	0.408	0.40	0.400	2.04
Median (IQR)	0.50 (0.44–0.55)	0.530 (0.463–0.562)	0.45 (0.45–0.45)	0.526 (0.461–0.560)	0.57 (0.18–0.70)	0.50 (0.33–0.50)	0.521 (0.461–0.650)	0.40 (0.40–0.40)	0.520 (0.460–0.649)	0.27 (0.11–0.61)

Ind.: Individual; Glob.: Global.

**Table 4:** Difference between individual and global fSampEn parameters in the cross-covariance of sEMG and |sMMG| fSampEn time-series and P_mo_ of COPD patients.

Subject	sEMG vs. P_mo_					|sMMG| vs. P_mo_				
	Ind. SD		Glob. SD		Δ*c_max_* (%)	Ind. SD		Glob. SD		Δ*c_max_* (%)
	Ind. *r*	*c_max_*	Glob. *r*	*c_max_*		Ind. *r*	*c_max_*	Glob. *r*	*c_max_*	
P1	0.20	0.649	0.20	0.648	0.10	0.60	0.376	0.50	0.377	0.22
P2	0.15	0.634	0.20	0.634	0.07	0.60	0.334	0.50	0.330	1.29
P3	0.15	0.555	0.20	0.545	1.72	0.60	0.425	0.50	0.425	0.11
P4	0.10	0.571	0.20	0.569	0.35	0.35	0.434	0.50	0.428	1.26
P5	0.15	0.822	0.20	0.822	0.00	0.60	0.551	0.50	0.550	0.29
P6	0.55	0.705	0.20	0.698	1.11	0.40	0.553	0.50	0.551	0.26
P7	0.40	0.756	0.20	0.753	0.32	0.60	0.630	0.50	0.632	0.24
P8	0.20	0.793	0.20	0.791	0.15	0.40	0.585	0.50	0.584	0.29
P9	0.50	0.665	0.20	0.659	0.82	0.20	0.247	0.50	0.233	5.75
P10	0.15	0.815	0.20	0.815	0.01	0.30	0.445	0.50	0.442	0.81
P11	0.30	0.794	0.20	0.793	0.11	0.60	0.523	0.50	0.524	0.21
P12	0.25	0.692	0.20	0.691	0.03	0.60	0.450	0.50	0.446	0.84
P13	0.15	0.628	0.20	0.627	0.19	0.50	0.554	0.50	0.553	0.14
P14	0.30	0.792	0.20	0.783	1.17	0.30	0.574	0.50	0.574	0.11
Median (IQR)	0.20 (0.15–0.30)	0.698 (0.638–0.793)	0.20 (0.20–0.20)	0.694 (0.637–0.789)	0.17 (0.08–0.7)	0.55 (0.36–0.60)	0.487 (0.427–0.553)	0.50 (0.50–0.50)	0.485 (0.426–0.552)	0.27 (0.21–0.83)

Ind.: Individual; Glob.: Global.
